# Does whole-body vibration training have a positive effect on balance and walking function in patients with stroke? A meta-analysis

**DOI:** 10.3389/fnhum.2022.1076665

**Published:** 2023-01-04

**Authors:** Yikun Yin, Jialin Wang, Zhengze Yu, Lina Zhou, Xiaoman Liu, Hejia Cai, Junzhi Sun

**Affiliations:** ^1^College of Physical and Health Education, Guangxi Normal University, Guilin, China; ^2^Institute of Sports Medicine and Health, Chengdu Sport University, Chengdu, China

**Keywords:** stroke, balance, walking function, whole-body vibration training, meta-analysis

## Abstract

**Objective:**

After a stroke, patients usually suffer from dysfunction, such as decreased balance ability, and abnormal walking function. Whole-body vibration training can promote muscle contraction, stimulate the proprioceptive system, enhance the muscle strength of low limbs and improve motor control ability. The study aims to evaluate the effectiveness of whole-body vibration training on the balance and walking function of patients with stroke.

**Methods:**

PubMed, CNKI, VIP, CBM, EBSCO, Embase and Web of Science were searched. According to the inclusion and exclusion criteria, randomized controlled trials on the effectiveness of whole-body vibration training on the balance and walking function of patients with stroke were collected. The search time ranged from the date of database construction to November 2022. The included trials were evaluated by the Cochrane risk-of-bias tool. The meta-analysis was performed using two software packages, consisting of RevMan 5.4 and Stata 12.2. If the results included in the literature were continuous variables, use the mean difference (MD) and 95% confidence interval (CI) for statistics.

**Results:**

(1) A total of 22 randomized controlled trials (RCTs) with a total of 1089 patients were included. (2) The results of meta-analysis showed that: compared with the controls, step length (MD = 6.12, 95%CI [5.63, 6.62], *p* < 0.001), step speed (MD = 0.14, 95%CI [0.09, 0.20], *p* < 0.001), cadence (MD = 9.03, 95%CI [2.23, 15.83], *p* = 0.009), stride length (MD = 6.74, 95%CI [−3.47, 10.01], *p* < 0.001), Berg Balance Scale (BBS) (MD = 4.08, 95%CI [2.39, 5.76], *p* < 0.001), Timed Up-and-Go test (TUGT) (MD = −2.88, 95%CI [−4.94, 0.81], *p* = 0.006), 10-meter Walk Test (10MWT) (MD = −2.69, 95%CI [−3.35, −2.03], *p* < 0.001), functional ambulation category scale (FAC) (MD = 0.78, 95%CI [0.65, 0.91], *p* < 0.001), Fugl-Meyer motor assessment of lower extremity (FMA-LE) (MD = 4.10, 95%CI [2.01, 6.20], *p* = 0.0001). (3) The results of subgroup analysis showed that, compared with other vibration frequencies, at 20–30 Hz frequency, WBV training had an obvious improvement effect only in TUGT. (4) The safety analysis showed that WBV training may be safe.

**Conclusion:**

Whole-body vibration training has a positive effect on the balance and walking function of patients with stroke. Thus, whole-body vibration training is a safe treatment method to improve the motor dysfunction of patients with stroke.

**Systematic review registration:**

[http://www.crd.york.ac.uk/PROSPERO], identifier [CRD4202348263].

## 1. Introduction

Stroke is one of the most prevalent cerebrovascular diseases. According to the most recent data, stroke is the second leading cause of death worldwide. With a high disability rate, recurrence rate, and fatality rate, stroke is also the most common cause of adult disability (Sacco and Rundek, 2012; Khaku and Tadi, 2022; Yikun et al., 2023). In the world each year, 16 million people get a stroke, according to a report issued by WHO in 2020. Recently, stroke incidence has been progressively rising with each passing year and is inclining to be younger along with the prolongation of the human life span (Guzik and Bushnell, 2017; Khaku and Tadi, 2022; Zhang et al., 2022). This is a formidable challenge for the medical and health systems (Moreno-Segura et al., 2022). The effects of a stroke on the human body vary depending on the degree of severity and location of the damage, but motor dysfunction, which manifests as decreased muscle strength, muscle spasms, abnormal muscle movement patterns, joint stiffness, abnormal proprioception, and other symptoms, is the most frequent symptom (Van Criekinge et al., 2019; Wei and Cai, 2022). The symptoms above would lead to decreased balance ability and abnormal walking function. After a stroke, more than 70% of patients experience varying degrees of lower limb dysfunction with limited recovery of walking function, resulting in most of them being unable to maintain a healthy gait or walking speed (Wist et al., 2016; Virani et al., 2020). The physical and psychological health of patients, as well as their quality of life and ability to reintegrate into family and society, are all significantly impacted by these dysfunctions. For the recovery of the walking function of stroke patients, the primary therapeutic methods at present include medication, muscle paste, PNF, rehabilitation training, machine exoskeletons, and so on (Wang et al., 2019; Varvarousis et al., 2021; Calafiore et al., 2022; Moreno-Segura et al., 2022; Nguyen et al., 2022).

Whole-body vibration (WBV) training helps to improve the dysfunction of the nervous system and musculoskeletal system diseases (In et al., 2018; Cigdem Karacay et al., 2022; Wang et al., 2022), to prevent and relieve osteoporosis in the elderly (Pichler et al., 2013; Cheng et al., 2021), and to promote sports injury recovery and improve sports performance (Sierra-Guzmán et al., 2018; Marin-Puyalto et al., 2020; Cheng et al., 2021). WBV training is a training method to improve neuromuscular, which uses mechanical vibration and external resistance load to stimulate the body to cause muscle vibration and increase the adaptiveness of the central nervous system (Choi et al., 2017). The patients sit or stand on the vibration platform, then the exogenous stimuli with various amplitudes and frequencies are transmitted from the platform to the whole body through the sole of the foot. The “bone-muscle-nerve” series connection is established (Jaime et al., 2019). By causing local or entire body muscles to vibrate, the vibration stimulation can increase the activation degree of the muscle spindle, cause high-frequency discharge and recruit more motor units, thus promoting muscle contraction, stimulating the proprioception system, enhancing muscle strength of lower limbs, and improving motor control ability (Marín et al., 2015; Liu et al., 2022).

The meta-analyses on WBV training interventions for stroke patients performed separately by Yang et al. (2015) and Lu et al. (2015) showed that WBV training has little role in improving balance and walking function in stroke patients. Yang and Butler (2020) concluded that controlled whole-body vibration training may benefit balance and mobility immediately, but the effects may not persist in stroke patients. Park et al. (2018) found that the effect sizes of WBV training for balance and gait function were small, through a comparison of effect improvement in all aspects of stroke patients after WBV intervention. After a collated analysis of researches above, we found that the databases searched were mainly Embase, PubMed, EBSCO, and Web of Science. The results and conclusions may be influenced by the inadequacy of the number of literature searches. In addition, the assessment methods for gait in the studies above mainly included TUGT, 10/6MWT and FAC, and no valid evaluation of walking spatiotemporal paraments (step length, step speed, and cadence, etc.) was performed. Therefore, in conclusion, the range of database searching was increased and the evaluation indicators of walking spatiotemporal paraments were added for analysis in this study.

Some studies have found that WBV training plays a positive role in the recovery of balance and walking function in patients with stroke (Gu and Hwangbo, 2016; Yan et al., 2021; Chuan et al., 2022), while other studies have shown that there is no significant difference between WBV training and routine rehabilitation training (Ijaz Ahmed Burq et al., 2021; Liu et al., 2022). The objective of this meta-analysis is to ascertain the effect of WBV training on balance and walking function in the rehabilitation of stroke patients, compared with routine, sham, and no treatments. Additionally, it sought to ascertain whether WBV training can serve as an effective training intervention method to guide clinical practice.

## 2. Materials and methods

### 2.1. Retrieval strategy

This meta-analysis was planned and implemented according to the Preferred Reporting Items for Systematic Reviews and Meta-Analyses (PRISMA) guidelines (Moher et al., 2015). The protocol was registered on the international prospective register of systematic reviews (http://www.crd.york.ac.uk/PROSPERO), registration number: CRD42022348263.

PubMed, CNKI, VIP, CBM, EBSCO, Embase and Web of Science were searched. The search time ranged from the date of database construction to November 2022. The last retrieval date is November 30, 2022. The literature search was conducted using a combination of subject terms and free terms. The search terms included “stroke,” “cerebral apoplexy,” “cerebral infarction,” “encephalorrhagia,” “walk,” “gait,” “progression,” “balance,” “whole-body vibration training,” “vibration training,” “vibration,” “VT,” “WBVT.” In order to get all the randomized control trials related to the whole-body vibration training intervention on the balance and walking function of patients with stroke, we also traced the references of the retrieved literature to supplement the relevant literature. The full search strategy for each database is presented in Supplementary material.

### 2.2. Literature inclusion, exclusion criteria and outcome indicator

The inclusion criteria: (1) Participants: stroke patients at any stage and time, regardless of sex, age, race and nationality; (2) Study design: randomized controlled trials (RCTs); (3) Primary treatment methods: vibration training alone or in combination with other treatments; (4) Treatment methods for the control group: any other interventions, including routine treatment, sham treatment, and no treatment; (5) Literature type: journal articles.

The exclusion criteria: (1) Literature not published in English or Chinese; (2) Literature published repeatedly; (3) Literature that was unable to effectively extract data and obtain original texts; (4) Animal studies or cross-sectional studies.

The primary outcome indicators: (1) walking spatiotemporal parament, consisting of step length (cm), step speed (m/s), cadence (step/min), single support time (s), double support time (s), stride length (cm) and step time (s). (2) Berg Balance Scale (BBS): BBS is a comprehensive scale to assess balance function in stroke patients, including the combined abilities of dynamic and static balance in sitting, standing and center of gravity movement. The scale is consisted of 14 items, each of which has a score of 0–4, with a maximum score of 56 points. The higher the score, the better the balance ability of the patient (ICC = 0.92) (Godi et al., 2013). (3) 10-meter Walk Test (10MWT): 10MWT is a commonly used measure for assessing dynamic walking function, which evaluating the time for patients to walk 10 meters at a natural pace (ICC = 0.96–0.98) (Peters et al., 2013). (4) Timed Up-and-Go test (TUGT): TUGT is a widely used performance test for the evaluation of coordination and stability in dynamic walking. TUGT requires participants to stand up from a chair, walk 3 meters, turn around, return to the chair, and sit down again (ICC > 0.95) (Hafsteinsdóttir et al., 2014).

The secondary outcome indicators: (1) Functional ambulation category scale (FAC): FAC is adopted to assess the walking ability of stroke patients. The test results of the scale were divided into 6 grades. The higher the grade, the better the walking ability (ICC = 0.95) (Mehrholz et al., 2007). (2) Fugl-Meyer motor assessment of lower extremity (FMA-LE): FAM-LE was conducted to evaluate the motor ability of lower limbs, containing contents from five domains (motion, sensation, balance, joint range of movement and pain) as well as 17 assessment items, with a full score of 34 (Gladstone et al., 2002).

### 2.3. Literature screening and data extraction

Step 1: Import retrieved literature to the literature management software EndNote X9.^1^ Step 2: Exclude duplicate materials. Step 3: Perform the first round of screening by reading titles and abstracts. Step 4: After downloading full texts, conduct a second round of screening to determine if inclusion criteria were met.

Two independent reviewers, ZY and LZ, conducted the literature screening and data extraction. Then a cross-checking was performed. When a possible disagreement occurred, we solved it through discussion or negotiation with a third independent reviewer, HC. In literature screening, we first read the title to exclude irrelevant literature, and then, we further read the abstract and the full text to determine whether to include it. If necessary, we would contact the author of the original research by email or telephone to obtain the unconfirmed information.

The extracted data: (1) General information of the included literature: the title, the first author and the year of publication; (2) General characteristics of the patients: the number of cases in each group, the age and the duration of the disease; (3) Treatment specifics and the follow-up time; (4) Key elements of bias risk assessment; (5) Focused outcome indicators.

### 2.4. Quality assessment

Two independent reviewers used the Cochrane Collaboration tool to examine the risk of bias for the included studies (Higgins et al., 2011; Cumpston et al., 2019), and cross-checking was conducted. A literature quality grade was performed according to the Jadad Scale. A score of 1–3 was considered low quality, and a score of 4–7 was considered high quality. The grading was also conducted by two independent reviewers, with the disagreement consulting the opinions of a third independent reviewer.

### 2.5. Statistical analysis

The statistical analysis was based on RevMan5.4 (the Review Manager software 5.4, The Nordic Cochrane Center, The Cochrane Collaboration). If the results included in the literature were continuous variables, use the mean difference (MD) and 95% confidence interval (CI) for statistics. The *p* value and the *I*^2^ index were used as indicators to assess the heterogeneity among studies. There was no heterogeneity between studies when *p* ≥ 0.10, while *p* < 0.10 indicates that there was heterogeneity between studies. The *I*^2^ index represented the degree of heterogeneity between studies. If *I*^2^ < 50%, it indicates that there was slight heterogeneity between the studies, and the fixed effect model was used for analysis. If *I*^2^ ≥ 50%, there was heterogeneity in the study, and the random effect model was used for analysis (Cochrane et al., 2021). The α value was set at 0.05 and Stata 12.0 software was used to conduct the publication bias analysis and sensitivity analysis of Begg’s test for the studies with more than 5 included outcome indicators. The threshold for statistical significance was set at *p* < 0.05.

Considering differences in WBV training frequencies, a subgroup analysis was conducted. When the vibration frequency was set at 20–50 Hz or 20–45 Hz, higher EMG activity was induced (Rittweger, 2010; Alam et al., 2018). Thus, muscle strength was enhanced and muscle training was more effective and several studies (Cardinale and Lim, 2003; Rittweger et al., 2003; Liu et al., 2022) have found vibration frequencies between 20 and 30 Hz to be more effective in stroke patients. Therefore, we planned to divide into two subgroups by frequency of WBV training: a subgroup of vibration frequencies at 20–30 Hz, and another subgroup of the other vibration frequencies.

And the safety analysis was also conducted to confirm the safety of WBV training, through the observed changes in blood pressure and heart rate or some terrible symptoms such as headache and nausea in stroke patients during WBV training in the included studies.

## 3. Results

**FIGURE 1:**
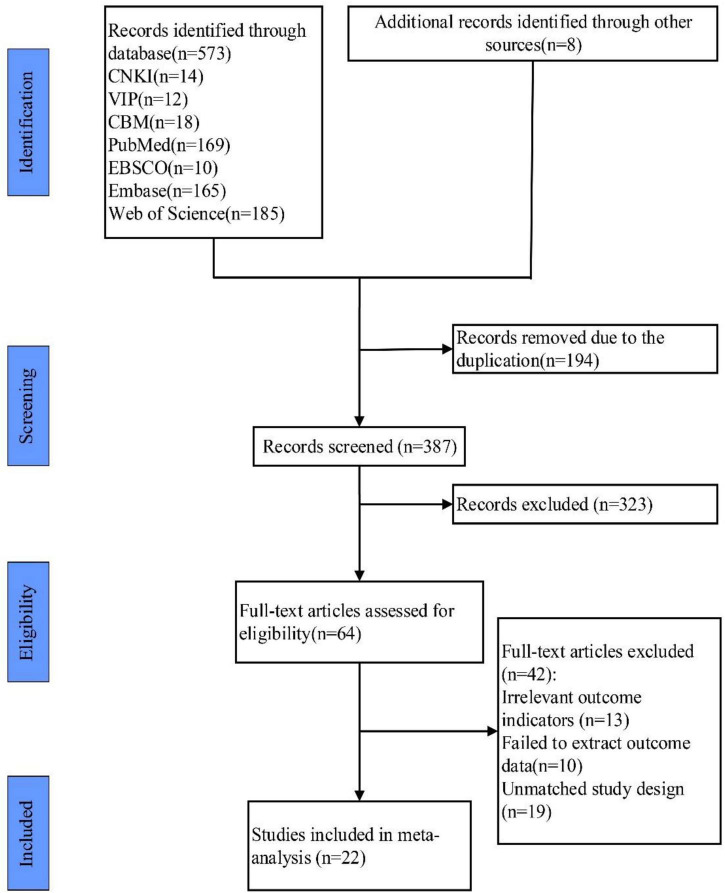
Study selection represented by PRISMA flowchart.

The initial search resulted in a total of 673 studies, and 8 studies were selected in other ways. EndNote X9 was used to remove duplicate documents, and there were 487 studies left. After reading the titles and abstracts, 122 studies were selected. Then, after reading the full texts, 98 studies were discarded because they did not meet the inclusion and exclusion criteria, and 22 studies were finally included (van Nes et al., 2006; Brogårdh et al., 2012; Chan et al., 2012; Guo et al., 2015; Choi et al., 2016, 2017; Gu and Hwangbo, 2016; Qianhao et al., 2018; Lee, 2019; Zhanyu et al., 2019; Zhen-hua et al., 2019; Sade et al., 2020; Xin-xin et al., 2020; Ijaz Ahmed Burq et al., 2021; Jin-Ming et al., 2021; Kim and Lee, 2021; Xie et al., 2021; Yan et al., 2021; Chuan et al., 2022; Le et al., 2022; Wei and Cai, 2022; Zhenying et al., 2022). The process is shown in Figure 1.

### 3.1. Characteristics of included studies

A total of 1,089 patients were included in the 22 studies. The age ranged from 31.8 to 78.31 years old. The sample size of each study ranged from 20 to 130 patients. 15 articles were published in the past five years, accounting for 68%. In most studies, the intervention in experimental groups was WVT combined with routine rehabilitation training. And in three studies, the experimental groups only used WVT intervention. In addition, the experimental groups in the other articles respectively added lower limb weight bearing training, basic walking training, extracorporeal shock wave therapy, music therapy, virtual reality technology, neuro-developmental treatment and treadmill training to routine rehabilitation training. For the control groups, in most studies, WVT was removed. However, there were five studies using the sham WVT intervention. The details of the research characteristics are shown in Table 1.

**TABLE 1:** The details of research characteristics.

No.	References	Sample size (Male/female, *N*)		Mean age, years		Course of disease		Description of intervention		Dosage	Outcome	Follow-up
		T	C	T	C	T	C	T	C			
1	Qianhao et al., 2018	9/6	11/4	49.33 ± 7.34	50.93 ± 8.19	60.73 ± 11.93 d	61.53 ± 11.10 d	F = 7 Hz, A = ± 4 mm and I	I	1 time/d, 10 min/time, 6 time/week, 4 weeks	①②④	No
2	Chuan et al., 2022	14/9	14/10	60.35 ± 11.63	62.42 ± 9.34	3.41 ± 1.48 m	3.23 ± 1.76 m	F = 5–8 Hz, A = 2 mm and I	I	1 time/d, 15 min/time, 5 time/week, 4 weeks	①②③④⑧⑨	No
3	Zhen-hua et al., 2019	18/17	16/19	55.0 ± 4.8	55.0 ± 4.7	63.7 ± 6.0 d	63.5 ± 5.0 d	F = 20 Hz, A = 3 mm and I, II	I,II	1 time/d, 5 time/week, 6 weeks	①②⑪	No
4	Xin-xin et al., 2020	16/4	15/5	55.15 ± 11.65	56.40 ± 10.92	2.60 ± 1.47 m	2.75 ± 1.77 m	F = 25 Hz, low A and I	I	1 time/d, 5 time/week, 4 weeks	③⑤⑥⑦	No
5	Yan et al., 2021	18/7	12/13	62.64 ± 7.02	61.92 ± 5.64	53.68 ± 8.88 d	53.28 ± 11.72 d	F = 12 Hz and I	I	1 time/d, 1 min/time, 5 time/week, 8 weeks	②⑦⑨	No
6	Zhanyu et al., 2019	9/6	8/7	48.1 ± 11.8	46.7 ± 10.9	82.5 ± 16 d	84.5 ± 17 d	F = 3–5 Hz and I, III	I,III	1 time/d, 15 min/time, 6 time/week, 8 weeks	④⑧⑩⑪	No
7	Le et al., 2022	13/12	14/11	63.53 ± 5.26	63.62 ± 4.21	130.35 ± 18.37 d	125.33 ± 20.32 d	A = 4 mm and I, IV	I,IV,V	1 time/d, 15 min/time, 5 time/week, 4 weeks	②③⑤⑦⑪	No
8	Jin-Ming et al., 2021	25/23	28/20	55.11 ± 4.36	54.90 ± 4.72	2.05 ± 0.89 m	2.06 ± 0.91 m	F = 12–20 Hz, A = 2.0–4.0 mm and I, VI	I	1 time/d, 9 min/time, 5 d/week, 6 weeks	⑦⑧⑩⑪	No
9	Zhenying et al., 2022	28/15	30/13	63.51 ± 7.56	62.67 ± 7.28	3.36 ± 1.52 m	3.76 ± 1.32 m	F = 20 Hz, A = 4 mm and I	I	1 time/d, 5 days/week, 8 weeks	①②⑦⑪	No
10	van Nes et al., 2006	16/11	14/12	59.7 ± 12.3	62.6 ± 7.6	38.9 ± 9.2 d	34.2 ± 11.1 d	F = 30 Hz, A = 3 mm and I, VII	I,V,VII	1 time/d, 20 min/time, 5 d/week, 6 weeks	⑦	Yes
11	Gu and Hwangbo, 2016	10	10	73.46 ± 3.94	73.46 ± 3.94	18.11 ± 13.04 m	18.11 ± 13.04 m	Vibration for 10 s and I	I	3 time/week, 16 min/time, 6 weeks	⑦⑧	No
12	Brogårdh et al., 2012	13/3	12/3	61.3 ± 8.5	63.9 ± 5.8	37.4 ± 31.8 m	33.1 ± 29.2 m	F = 25 Hz, A = 3.75 mm	V	2 time/week, each time<45 min, 6 weeks	⑦	No
13	Wei and Cai, 2022	L: 23/3 H: 21/5	21/5	L: 72.42 ± 5.89 H: 70.19 ± 5.07	71.85 ± 6.03	L: 33.65 ± 15.75 m H: 36.69 ± 20.32 m	31.23 ± 19.33 m	L:F = 13 Hz and I H: F = 26 Hz and I	I,V	5 d/week, 6 min/time, 5 weeks	⑦	No
14	Ijaz Ahmed Burq et al., 2021	21/11	19/13	54.56 ± 8.85	55.06 ± 11.48	14.46 ± 12.14 m	10.82 ± 5.15 m	F = 120 Hz and I	I	6 d/week, 15 min, 2 weeks	⑧⑨	No
15	Chan et al., 2012	10/5	11/4	56.07 ± 11.04	54.93 ± 7.45	30.40 ± 25.80 m	38.87 ± 38.22 m	F = 12 Hz, A = 4 mm	V	20 min, a single time	③⑧⑨	no
16	Sade et al., 2020	14/12	9/8	46.8 ± 15	51.6 ± 10	34.5 ± 25 m	35.5 ± 20 m	F = 35–40 Hz, A = 2 mm and I	I	10 min, 3 weeks	①②③④⑥⑦⑧	no
17	Choi et al., 2016	8/3	7/4	50.9 ± 8.2	52.2 ± 12.3	12.3 ± 10.1 y	10.6 ± 6.8 y	F = 25 Hz, A = 5 mm and VIII	VIII	5 d/week, 10 min, 4 weeks	⑧	no
18	Kim and Lee, 2021	8/12	7/11	57.20 ± 11.00	55.70 ± 10.40	31.60 ± 15.18 d	28.00 ± 8.72 d	F = 16 Hz and I	I	5 d/week, 20 min, 2 weeks	⑦⑧⑨⑩	no
19	Choi et al., 2017	8/7	11/4	51.93 ± 8.35	53.67 ± 7.38	25.13 ± 9.25 m	22.53 ± 10.27 m	F = 5–30 Hz (add 5 Hz each 2 weeks), A = 3mm and IX	IX	3 time/week, 6 weeks	①②③⑤	no
20	Lee, 2019	6/3	8/4	59.78 ± 5.78	61.25 ± 10.06	84.11 ± 10.76 m	98.42 ± 22.76	F = 116 Hz, A = 3 mm and I	I	3 d/week, 30 min, 6 weeks	①②③④⑤	no
21	Guo et al., 2015	15	15	53.8 ± 6.0	54.3 ± 6.8	66.9 ± 42.9 d	59.4 ± 61.4	F = 6 (1–2 w), 8 (3–5 w), 10 (6–8 w) Hz; A = 4 mm	V	8 weeks	⑨⑪	no
22	Xie et al., 2021	32/33	33/32	60.42 ± 6.39	59.82 ± 6.62	3.22 ± 1.35 m	3.09 ± 1.01 m	F = 20 Hz, A = 5.2 mm and I	I	6 d/week, 22 min, 4 weeks	⑦⑧⑨	no

T, experimental groups; C, control groups; I, routine rehabilitation training (active and passive limb activities, muscle strength training, neuromuscular facilitation techniques, balance training, and physical factor therapy etc.); II, weight bearing training of affected lower limb; III, basic walking training; IV, extracorporeal shock wave therapy; V, sham WBV training; VI, virtual reality technology; VII, music therapy; VIII, neuro-developmental treatment; IX, treadmill training. ① step length; ② step speed; ③ cadence; ④ single support time/double support time; ⑤ stride length; ⑥ step time; ⑦ Berg Balance Scale (BBS); ⑧ Timed Up-and-Go test (TUGT); ⑨ 10-meter Walking Test (10MWT); ⑩ functional ambulation category scale (FAC); ⑪ Fugl-Meyer motor assessment of lower extremity (FMA-LE). F, frequency; A, amplitude; L, low-frequency group; H, high-frequency group; d, day; m, month; y, year.

In 11 of the included studies, the walking spatiotemporal parament was evaluated (step length, step speed, cadence, single support time, double support time, stride length and step times). In 13 of the included studies, Berg Balance Scale (BBS) was used. And TUGT, 10MWT, FAC, and FMA-L were respectively used in 10, 7, 4 and 4 studies.

**FIGURE 2:**
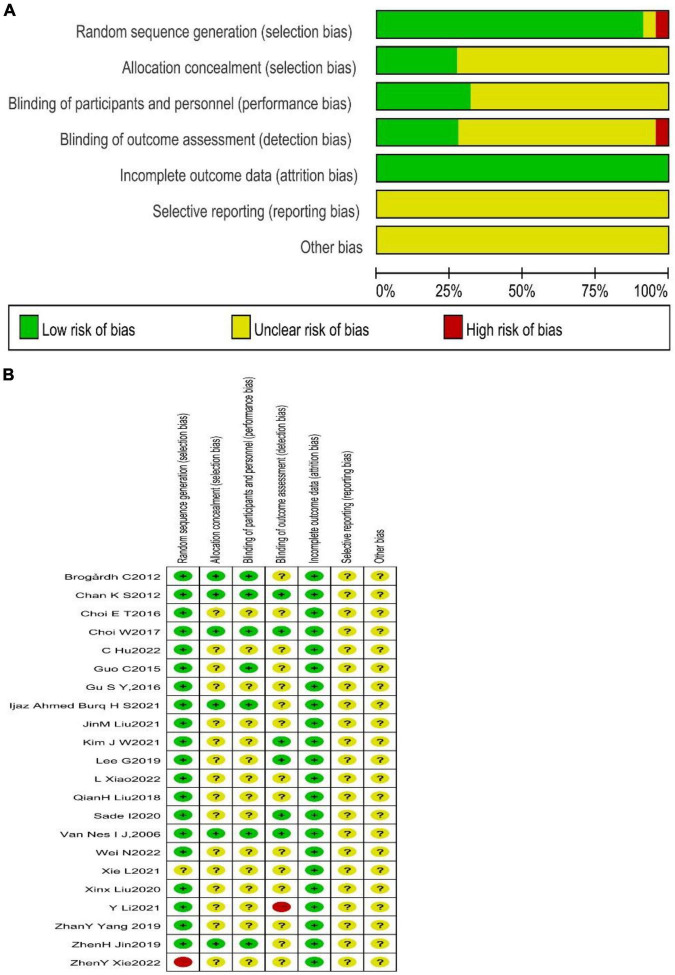
The risk assessment of bias by Cochrane. **(A)** This figure summarized the quality assessment of all included studies and presented an overall study quality assessment and risk of bias. Green means low risk of bias, red means high risk of bias, and yellow means ominous risk of bias. **(B)** This figure was presented separately for each included study and intuitively presented the quality assessment and risk of bias of each study. “+” Means low risk of bias, “–” means high risk of bias, and “?” means unknown risk of bias.

**FIGURE 3:**
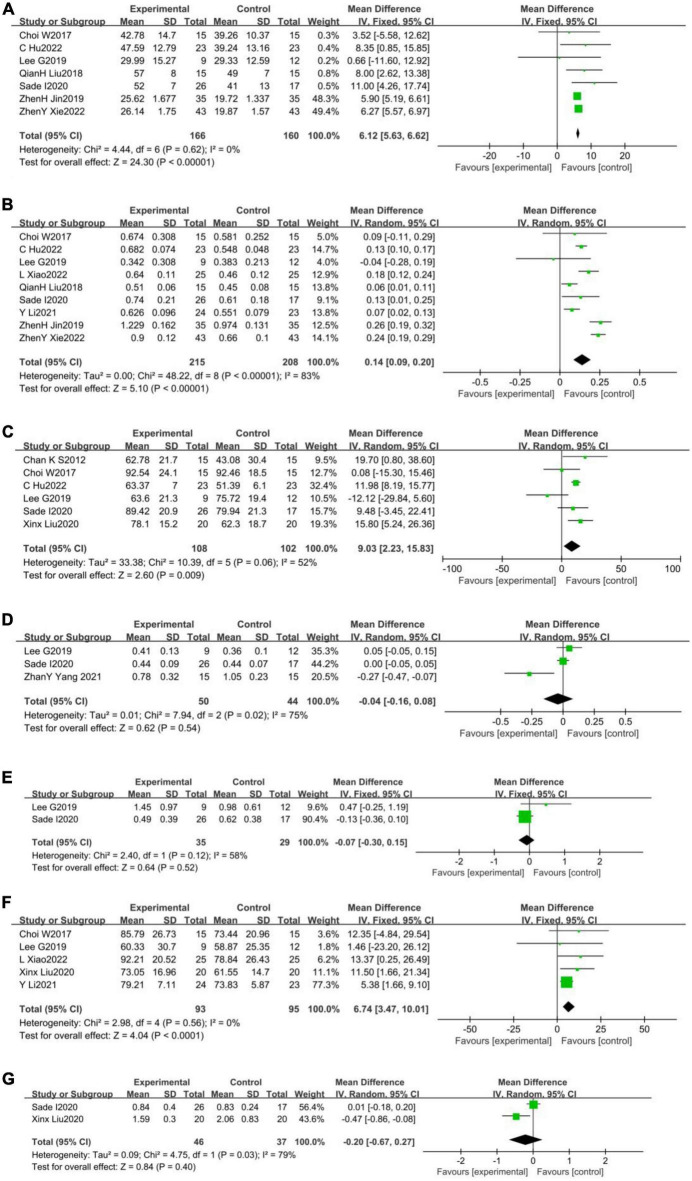
Forest plot of the effects of WBV training on walking spatiotemporal parament. **(A)** Step length (cm); **(B)** step speed (m/s); **(C)** cadence (step/min); **(D)** single support time(s); **(E)** double support time (s); **(F)** stride length (cm); and **(G)** step time (s).

The risk of bias assessment was performed through RevMan5.4 software, according to the Cochrane Handbook for Systematic Reviews. The results are shown in Figures 2, 3. The quality of the literature was graded according to the Jadad scale, with two studies judged to be of low quality and the remaining studies considered to be of high quality. The details are presented in Supplementary material.

### 3.2. Results of the meta-analysis

#### 3.2.1. Walking spatiotemporal parament

A total of seven studies (Choi et al., 2017; Qianhao et al., 2018; Lee, 2019; Zhen-hua et al., 2019; Sade et al., 2020; Chuan et al., 2022; Zhenying et al., 2022) reported the effect of WBV training on step length in stroke patients, with non-heterogeneity among them (*p* = 0.62, *I*^2^ = 0%). A fixed effects model was used to analyze the data (MD = 6.12, 95%CI [5.63, 6.62], *p* < 0.001; Figure 3A). A total of nine studies (Choi et al., 2017; Qianhao et al., 2018; Lee, 2019; Zhen-hua et al., 2019; Sade et al., 2020; Yan et al., 2021; Chuan et al., 2022; Le et al., 2022; Zhenying et al., 2022) reported the effect of WBV training on step speed in stroke patients, with substantial heterogeneity among them (*p* < 0.001, *I*^2^ = 83%). A random effects model was used to analyze the data (MD = 0.14, 95%CI [0.09, 0.20], *p* < 0.001) (Figure 3B). A total of six studies (Chan et al., 2012; Choi et al., 2017; Lee, 2019; Sade et al., 2020; Xin-xin et al., 2020; Chuan et al., 2022)reported the effect of WBV training on the cadence in stroke patients, with substantial heterogeneity among them (*p* = 0.06, *I*^2^ = 52%). A random effects model was used to analyze the data (MD = 9.03, 95%CI [2.23, 15.83], *p* = 0.009) (Figure 3C). A total of three studies (Lee, 2019; Zhanyu et al., 2019; Sade et al., 2020) reported the effect of WBV training on the single support time in stroke patients, with substantial heterogeneity among them (*p* = 0.02, *I*^2^ = 75%). A random effects model was used to analyze the data (MD = −0.04, 95%CI [−0.16, 0.08], *p* = 0.54) (Figure 3D). There were only two studies (Lee, 2019; Sade et al., 2020) reported the effect of WBV training on the double support time in stroke patients, with substantial heterogeneity among them (*p* = 0.12, *I*^2^ = 58%). A random effects model was used to analyze the data (MD = −0.07, 95%CI [−0.30, 0.15], *p* = 0.52) (Figure 3E). A total of five studies (Choi et al., 2017; Lee, 2019; Xin-xin et al., 2020; Yan et al., 2021; Le et al., 2022) reported the effect of WBV training on the stride length in stroke patients, with non-heterogeneity among them (*p* = 0.56, *I*^2^ = 0%). A fixed effects model was used to analyze the data (MD = 6.74, 95%CI [−3.47, 10.01], *p* < 0.001) (Figure 3F). Finally, a total of two studies (Sade et al., 2020; Xin-xin et al., 2020)reported the effect of WBV training on the step time in stroke patients, with substantial heterogeneity among them (*p* = 0.03, *I*^2^ = 79%). A random effects model was used to analyze the data (MD = −0.20, 95%CI [−0.67, 0.27], *p* = 0.40) (Figure 3G).

#### 3.2.2. Berg Balance Scale (BBS)

**FIGURE 4:**
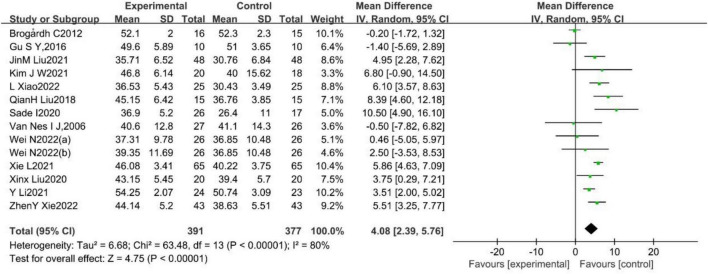
Forest plot of the effects of WBV training on BBS.

A total of 13 studies (van Nes et al., 2006; Brogårdh et al., 2012; Gu and Hwangbo, 2016; Qianhao et al., 2018; Sade et al., 2020; Xin-xin et al., 2020; Jin-Ming et al., 2021; Kim and Lee, 2021; Xie et al., 2021; Yan et al., 2021; Le et al., 2022; Wei and Cai, 2022; Zhenying et al., 2022)reported the effect of WBV training on BBS in stroke patients, with substantial heterogeneity among them (*p* < 0.001, *I*^2^ = 80%). A random effects model was used to analyze the data (MD = 4.08, 95%CI [2.39, 5.76], *p* < 0.001; Figure 4).

#### 3.2.3. Timed Up-and-Go test (TUGT) (s)

**FIGURE 5:**
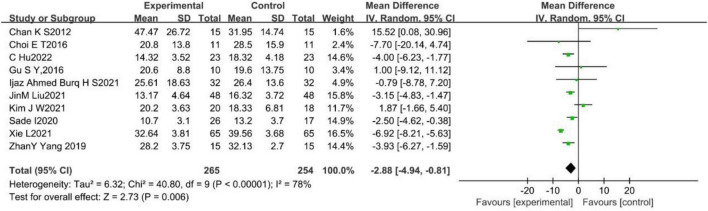
Forest plot of the effects of WBV training on TUGT.

A total of 10 studies (Chan et al., 2012; Choi et al., 2016; Gu and Hwangbo, 2016; Sade et al., 2020; Ijaz Ahmed Burq et al., 2021; Jin-Ming et al., 2021; Kim and Lee, 2021; Xie et al., 2021; Chuan et al., 2022; Zhenying et al., 2022) reported the effect of WBV training on TUGT in stroke patients, with substantial heterogeneity among them (*p* < 0.001, *I*^2^ = 78%). A random effects model was used to analyze the data (MD = −2.88, 95%CI [−4.94, −0.81], *p* = 0.006; Figure 5).

#### 3.2.4. 10-meter Walk Test (10MWT) (s)

**FIGURE 6:**
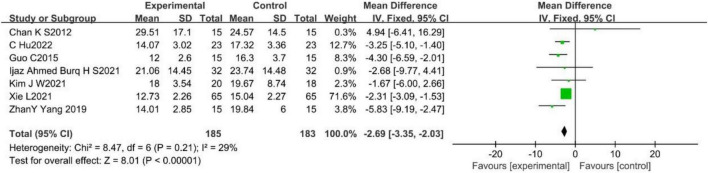
Forest plot of the effects of WBV training on 10MWT.

A total of seven studies (Chan et al., 2012; Guo et al., 2015; Zhanyu et al., 2019; Ijaz Ahmed Burq et al., 2021; Kim and Lee, 2021; Xie et al., 2021; Chuan et al., 2022) reported the effect of WBV on 10MWT in stroke patients, with no-heterogeneity among them (*p* = 0.21, *I*^2^ = 29%). A fixed effects model was used to analyze the data (MD = −2.69, 95%CI [−3.35, −2.03], *p* < 0.001; Figure 6).

#### 3.2.5. Functional ambulation category scale (FAC)

**FIGURE 7:**
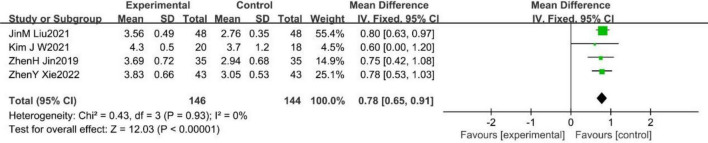
Forest plot of the effects of WBV training on FAC.

A total of four studies (Zhen-hua et al., 2019; Jin-Ming et al., 2021; Kim and Lee, 2021; Zhenying et al., 2022) reported the effect of WBV training on FAC in stroke patients, with no heterogeneity among them (*p* = 0.93, *I*^2^ = 0%). A fixed effects model was used to analyze the data (MD = 0.78, 95%CI [0.65, 0.91], *p* < 0.001; Figure 7).

#### 3.2.6. Fugl-Meyer motor assessment of lower extremity (FMA-LE)

**FIGURE 8:**
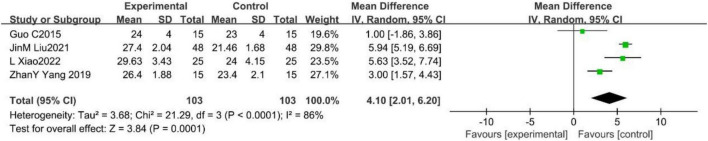
Forest plot of the effects of WBV training on FMA-LE.

A total of four studies (Guo et al., 2015; Zhanyu et al., 2019; Jin-Ming et al., 2021; Le et al., 2022) reported the effect of WBV training on FMA-LE in stroke patients, with substantial heterogeneity among them (*p* < 0.001, *I*^2^ = 86%). A random effects model was used to analyze the data (MD = 4.10, 95%CI [2.01, 6.20], *p* = 0.0001; Figure 8).

In summary, the results showed that the improvements in step length, step speed, cadence, stride length, BBS, TUGT, 10MWT, FAC and FAM-LE in the experimental groups were better than those in the controls, and the improvements in single support time, double support time and step time in the experimental groups were not obviously better than those in the controls.

### 3.3. Subgroup analysis

It had been suggested that the frequency of WBV training set from 20 to 30 Hz may be more beneficial in stroke patients (Cardinale and Lim, 2003; Rittweger et al., 2003; Liu et al., 2022). Thus, a subgroup analysis was conducted. The results showed that a 20–30 Hz vibration frequency was not conducive to the improvement of step length, BBS and FAC. However, in terms of the results of TUGT, the 20–30 Hz vibration frequency was better than other frequencies. The details are presented in Supplementary Figures 1–4.

### 3.4. Publication bias and sensitivity analysis

The publication bias analysis was conducted for the outcome indicators included in five or more studies through Begg’s test. The results showed step length (*t* = 0.44, *P* = 0.681, *P* > 0.05), step speed (*t* = −2.14, *P* = 0.07, *P* > 0.05), cadence (*t* = −1.11, *P* = 0.328, *P* > 0.05), BBS (*t* = 1.43, *P* = 0.180, *P* > 0.05), TUGT (*t* = 1.56, *P* = 0.157, *P* > 0.05) and 10MWT (*t* = 1.61, *P* = 0.151, *P* > 0.05), indicating no significant publication bias. The details are presented in Supplementary Figures 5–11.

A sensitivity analysis was conducted on the results of the meta-analysis with substantial heterogeneity and significant differences through one-by-one elimination. The results showed that there was no heterogeneity after excluding two studies (Zhen-hua et al., 2019; Zhenying et al., 2022) in the sensitivity analysis for the indicator of step length. The heterogeneity may be caused by the different measurement methods for the step length, with respectively using the footprint measurement method and gait analysis system in the two studies. In addition, in the sensitivity analysis for the indicators of step speed, cadence, stride length, BBS, TUGT and 10MWT, there was no obvious change when excluding any one of the studies. It indicated that the result was robust.

### 3.5. Safety analysis

Safety and adverse reactions were mentioned in three studies (Sade et al., 2020; Chuan et al., 2022; Wei and Cai, 2022). No adverse reactions occurred in the study. In general, the whole-body vibration training was safe.

## 4. Discussion

After a stroke, patients usually suffered from decreased muscle strength, abnormal muscle tension, limb coordination disorder, and sensory abnormality, caused by the injury of upper motor neurons (Zhen-hua et al., 2019), leading to the decline of gait stability, which will seriously affect the lower limb balance and walking function. For 85% of patients with stroke, the primary rehabilitation target is the recovery of walking function (Candelise et al., 2007), reducing the time required for patients to return to family and society, and improving their quality of life. Therefore, the main goal of the treatment is to improve gait stability and enhance the walking function of stroke patients.

WBV training has been widely used to promote the rehabilitation of stroke patients, and its effectiveness in the recovery of dysfunction in neurological diseases has been demonstrated to a certain degree (Alizadeh-Meghrazi et al., 2014; Kim and Lee, 2021; Tekin and Kavlak, 2021). However, previous studies may have been limited by insufficient literature searches and evaluation indicators. And a small part of the research showed that WBV had no positive effect on balance and gait improvement in stroke patients (Ijaz Ahmed Burq et al., 2021; Liu et al., 2022). Therefore, according to the disadvantage in the previous research, we added the search range of the database and evaluation indicators of walking spatiotemporal paraments to the analysis. And 22 randomized controlled trials (RCTs), which were called studies with the highest reliability and quality of research data (Barton, 2000), were finally included. In addition, adverse events were also considered because safety was an important component of treatments.

The decreased muscle strength in the lower limbs is a major cause of the unhealthy gait in stroke patients (Lau et al., 2011). The muscle strength in lower limbs was positively associated with spatiotemporal variations in stride length, stride time, stance time and double support time in old adults (Abdul Jabbar et al., 2021). The meta-analysis revealed that different changes in walking spatiotemporal parameters occurred after the intervention of WBV training for stroke patients. The improvement of step length, step speed, cadence and stride length was significant (*p* < 0.05). The impact stimulation produced in WBV training transfers to the whole body, stimulates proprioception receptors such as muscle spindles and tendon spindles, increases the excitability of sensory nerve fiber endings, and induces the stretch reflex in skeletal muscle. Therefore, the degree of muscle activation is increased, and the latent motor units are further activated. In this way, muscles recruit more motor units (Fallon and Macefield, 2007; Miyara et al., 2022). The improvement of muscle recruiting efficiency is beneficial to enhance the strength of muscles and improve sports performance (Sohrabzadeh et al., 2022). Several studies (Lau et al., 2011; Sitjà-Rabert et al., 2012; Alam et al., 2018) have shown that WBV training can enhance electromyography (EMG) signals in the lower limbs, improve muscle activation, and increase muscle strength, flexibility, burst force, and balance ability in the elderly. WBV training can also improve musculoskeletal health by increasing the production of fibronectin type III domain-containing protein 5 (FNDC5) and regulating the expression of key markers like myostatin, which has effects on both muscle and bone tissue (Cariati et al., 2022). Furthermore, the meta-analysis revealed that WBV training improved single support time, double support time, and step time, but not significantly (*p* > 0.05) when compared to controls. Stroke patients mostly suffer from damage to one cerebral hemisphere, which leads to dysfunction of the contralateral limb (Sacco and Rundek, 2012). Therefore, there was a large variation in the amplitude of the left-right movement of the body’s center of gravity during walking. To provide adequate stability, the unaffected side requires greater autonomic control (Song et al., 2018). Besides that, because of abnormal proprioception in stroke patients, the compensatory phenomenon occurs during body postural control, which then triggers symptoms such as abnormal gait posture (Dingwell and Cavanagh, 2001). Song S et al. found that an 8-week WBV training could improve the sensation, stability, and motility in the feet and ankles, with single support time and double support time decreasing (Song et al., 2018). This is inconsistent with the results of the meta-analysis. The different intervention cycles could be the cause of this occurrence. The WBV training intervention cycle was 3 or 4 weeks in the included literature in the meta-analysis.

The meta-analysis showed that WBV training had an obvious positive effect on balance, walking function and dynamic stability in stroke patients. The vibration could influence the activity rhythm of spinal anterior horn neurons, activate the cerebral motor cortex excitability, promote the remodeling of neural function in injured regions, and improve the postural control ability of patients with stroke (Mouchnino and Blouin, 2013; Lapole et al., 2015; Jammes et al., 2018). The vibration stimulus will induce the presynaptic inhibition of Ia afferents and/or a neurotransmitter depletion in presynaptic terminals. This may decrease the monosynaptic reflex excitability, reduce the abnormal spinal reflex excitation, restrain the stretch and H reflexes of the muscles, and regulate muscle spasm. Therefore, the balance and walking function of patients with stroke were improved (Miyara et al., 2014; Gu and Hwangbo, 2016). And studies (Kipp et al., 2011; Kim and Lee, 2021), showed that amplitudes of H reflexes of the soleus and gastrocnemius in the lower leg were significantly reduced after WBV training in patients with stroke. The main feature of WBV training, as a relatively passive exogenous stimulus, is effective training achieved with a smaller load intervention, which reduces the cardiopulmonary burden, compared with other rehabilitation training methods (Muir et al., 2013). WBV training has also been shown to increase oxygen consumption by itself in stroke patients and to promote the release of vasodilators without additional effects on heart rate or blood pressure (Zhang et al., 2022). And the vibration stimulation can promote osteoblast differentiation and subsequently osteogenesis and increase bone mass by activating the Wnt signaling pathway of bone marrow stromal cells (Yu-Han et al., 2013), thereby preventing and alleviating osteoporosis in the elderly (Cheng et al., 2021).

The subgroup analysis revealed that, after WBV training at 20–30 Hz, only the improvement of TUGT was better than at other frequencies, while that of step length, BBS and FAC was not. It has been found that, even for healthy individuals, early muscle fatigue is induced when the WBV vibration frequency exceeds 30 Hz. And for patients with stroke, a similar situation will happen when the WBV training frequency is set at equal to or less than 30 Hz with a 3 mm amplitude (van Nes et al., 2006; Tihanyi et al., 2007; Rittweger, 2010; Pujari et al., 2019). It has also been found that WBV training at a frequency of 20–45 Hz produces a positive muscle training effect (Rittweger, 2010). And when the WBV training frequency is set from 20 to 50 Hz, the variation of EMG signals is great, thereby enhancing muscle strength (Alam et al., 2018). However, at present, there is no guideline for recommended frequencies and amplitudes of WBV training for patients with stroke. It is suggested that the research should focus on this, to identify a suitable treatment strategy for stroke patients.

WBV training can induce the expression of brain-derived neurotrophic factor (BDNF) and FNDC5 in the cerebellum and hippocampus of the mouse to stimulate learning ability and cognitive memory (Cariati et al., 2021). And WBV training can improve brain health and cognitive function, as well as slow the problem of muscle wasting and motor decline associated with aging and/or a sedentary lifestyle (Cariati et al., 2022). Therefore, in future studies, it is possible to explore the effects of WBV training on cognitive impairment in stroke patients or those with other neurological injuries, and further explore the potential physiological and molecular mechanisms of WBV training.

## 5. Conclusion and suggestion

The meta-analysis revealed that whole-body vibration training has a positive effect on the balance and walking function of patients with stroke. Whole-body vibration training is also a safe treatment method for recovering from the walking dysfunction of patients with stroke.

Due to the fewer follow-ups in the included RCTs, the lack of long-term treatment effect observation and the differences in intervention protocols, the results of the included RCTs were insufficiently consistent. Thus, further studies for the conclusion of the meta-analysis are required. It is suggested that a more unified and standardized research design and intervention protocol should be established in future studies, while the potential mechanism of the impact of the intensity and physical parameters of WBV training on the efficacy of stroke patients should be further researched. And, to further verify the authenticity of the efficacy, objective instruments should be added to evaluate the indicator such as walking spatiotemporal parament.

## 6. Limitation of meta-analysis

1) The included studies were only in two languages: Chinese and English. And the sample size of the included studies was relatively small, which may have made the results biased.
2) Overall, the heterogeneity among some studies was substantial, which may have had effects on the reliability of the meta-analysis.
3) The vibration frequency, amplitude and time of the included studies were not sufficiently identical. And the intervention methods for the controls were also not identical enough. Therefore, these may influence the accuracy of the results of the meta-analysis.
4) The difference in the measurement method of the walking spatiotemporal parament may result in a measurement bias, thus affecting the analytical results.

## Data availability statement

The original contributions presented in this study are included in the article/Supplementary material, further inquiries can be directed to the corresponding author.

## Author contributions

YY and JS designed the systematic review and supervised the entire program. JW, HC, LZ, and XL reviewed all the studies and extracted the information from the eligible trials. YY and JW analyzed the data and prepared the figures and table. YY, JW, and ZY wrote the manuscript. YY, ZY, and JS revised the manuscript. All authors reviewed and approved the manuscript.

## Footnotes

1
www.endnote.com
