# Supplementary material for: Does whole-body vibration training have a positive effect on balance and walking function in patients with stroke? A meta-analysis
Source: Front Hum Neurosci. 2023 Jan 4;16:1076665. doi: 10.3389/fnhum.2022.1076665 (PMC9846107; doi:10.3389/fnhum.2022.1076665)
Supplement: Supplementary file 1 [file Data_Sheet_1.docx]

Supplementary Material

# Supplementary Figures and Tables

## Supplementary Figures


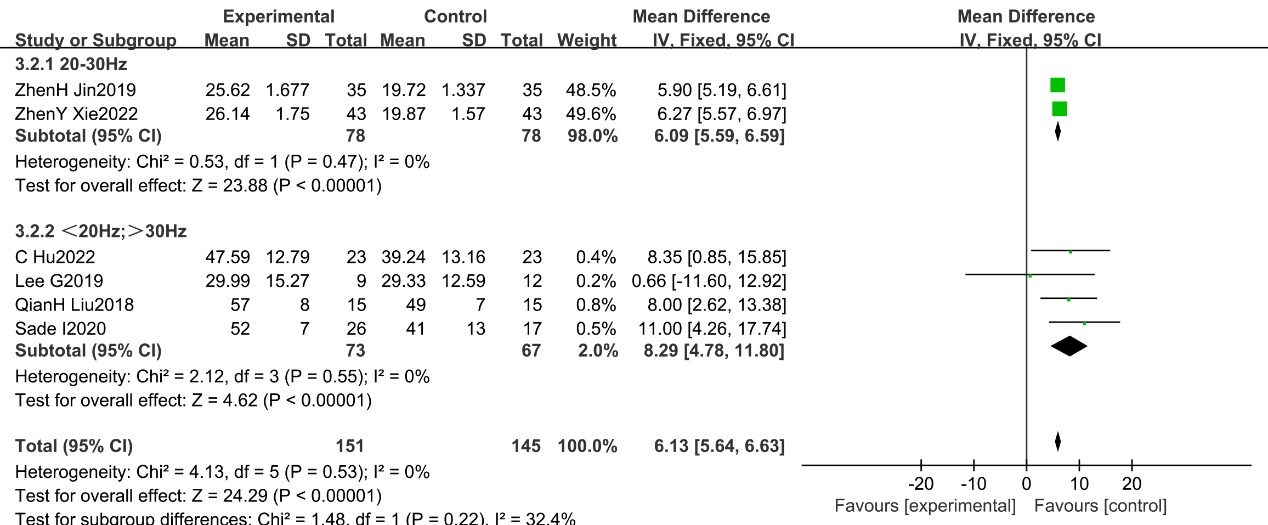


Fig S1 Subgroup analysis of the effect of different frequencies on step length in stroke patients


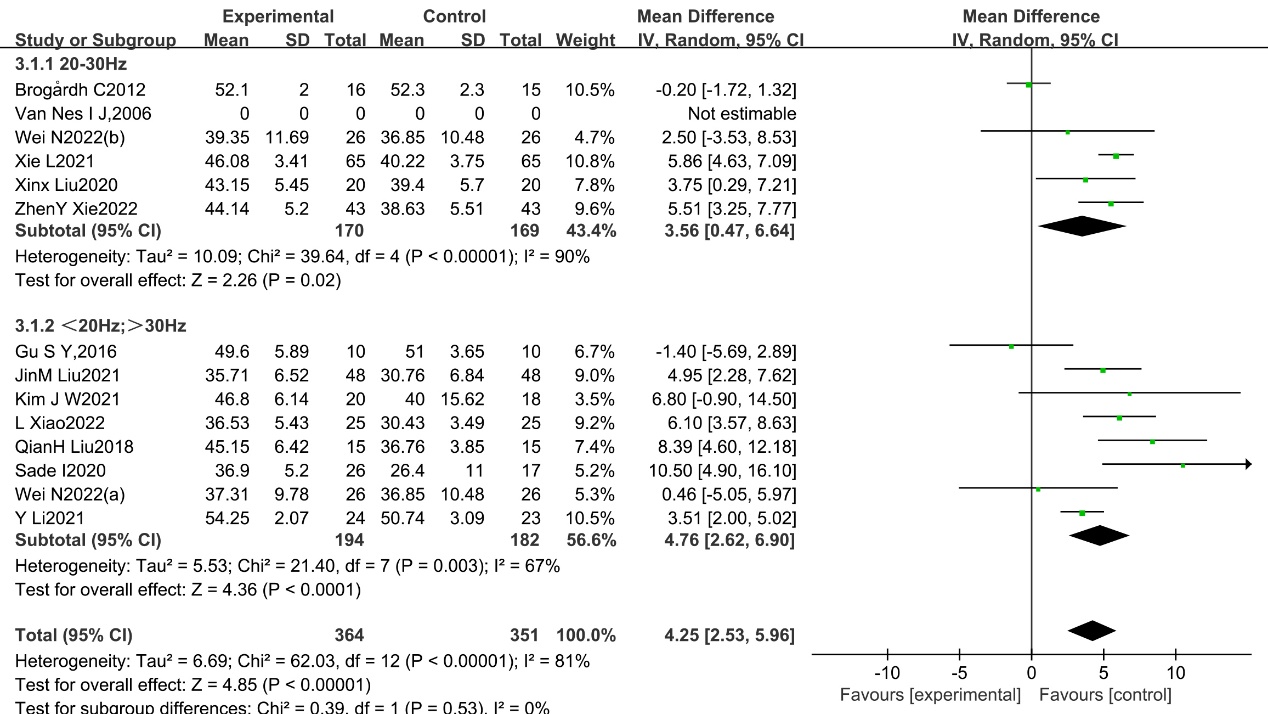


Fig S2 Subgroup analysis of the effect of different frequencies on BBS in stroke patients


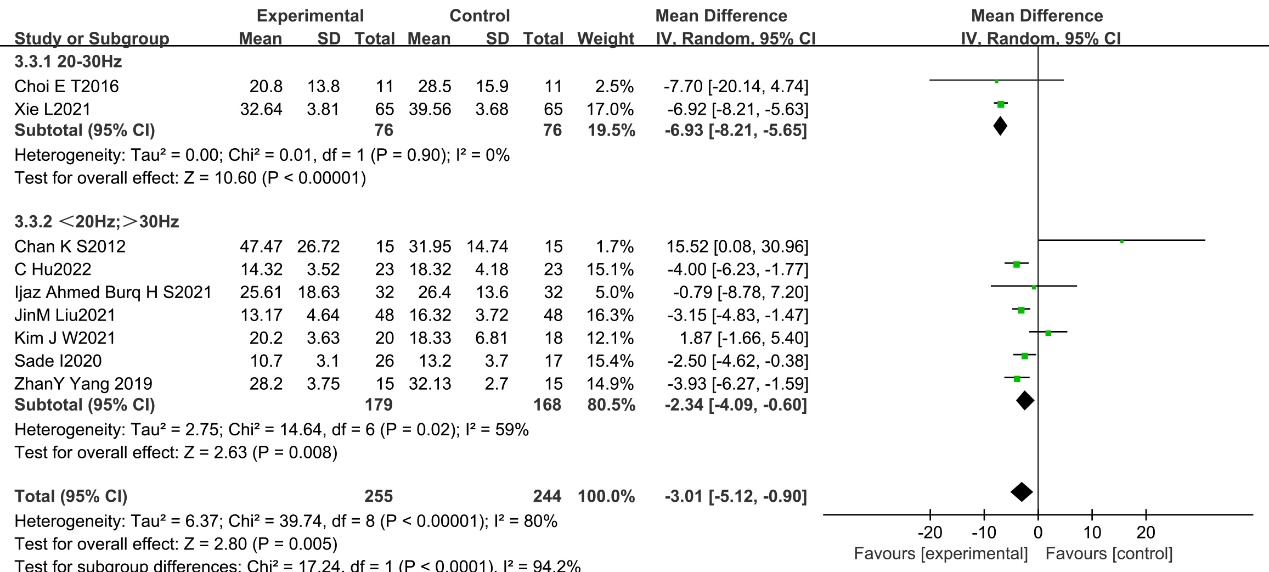


Fig S3 Subgroup analysis of the effect of different frequencies on TUGT in stroke patients


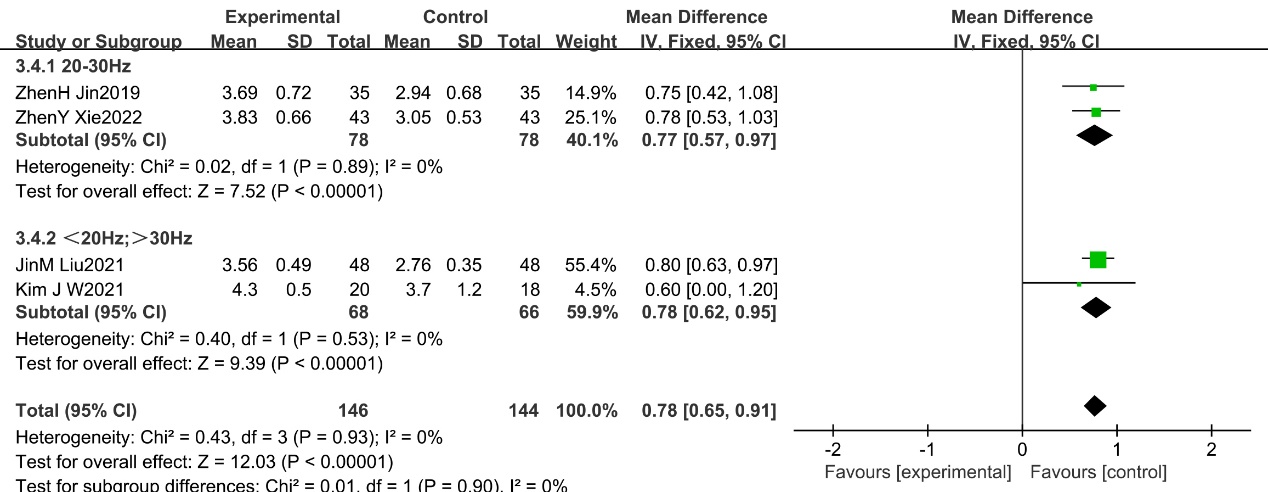


Fig S4 Subgroup analysis of the effect of different frequencies on FAC in stroke patients


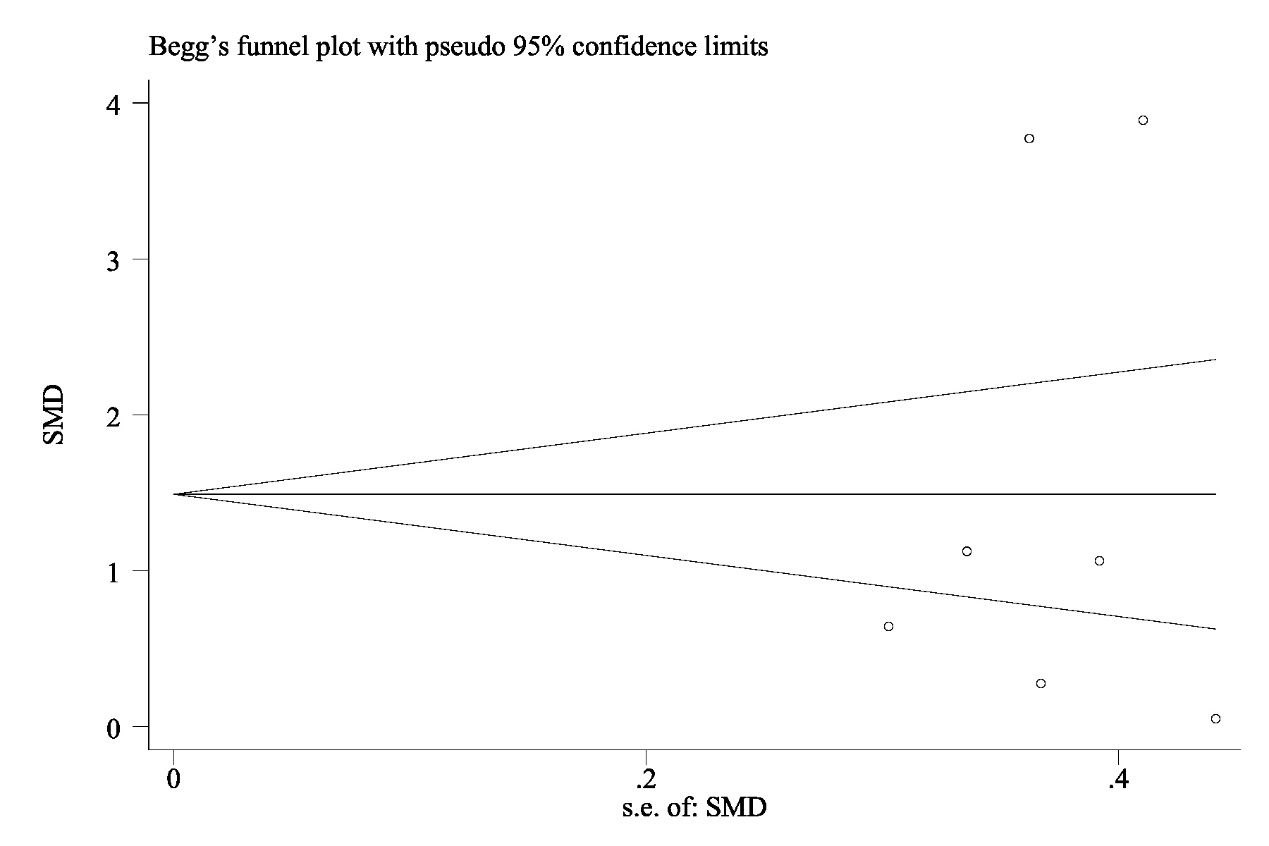


Fig S5 Step Length publication bias figure of included literature


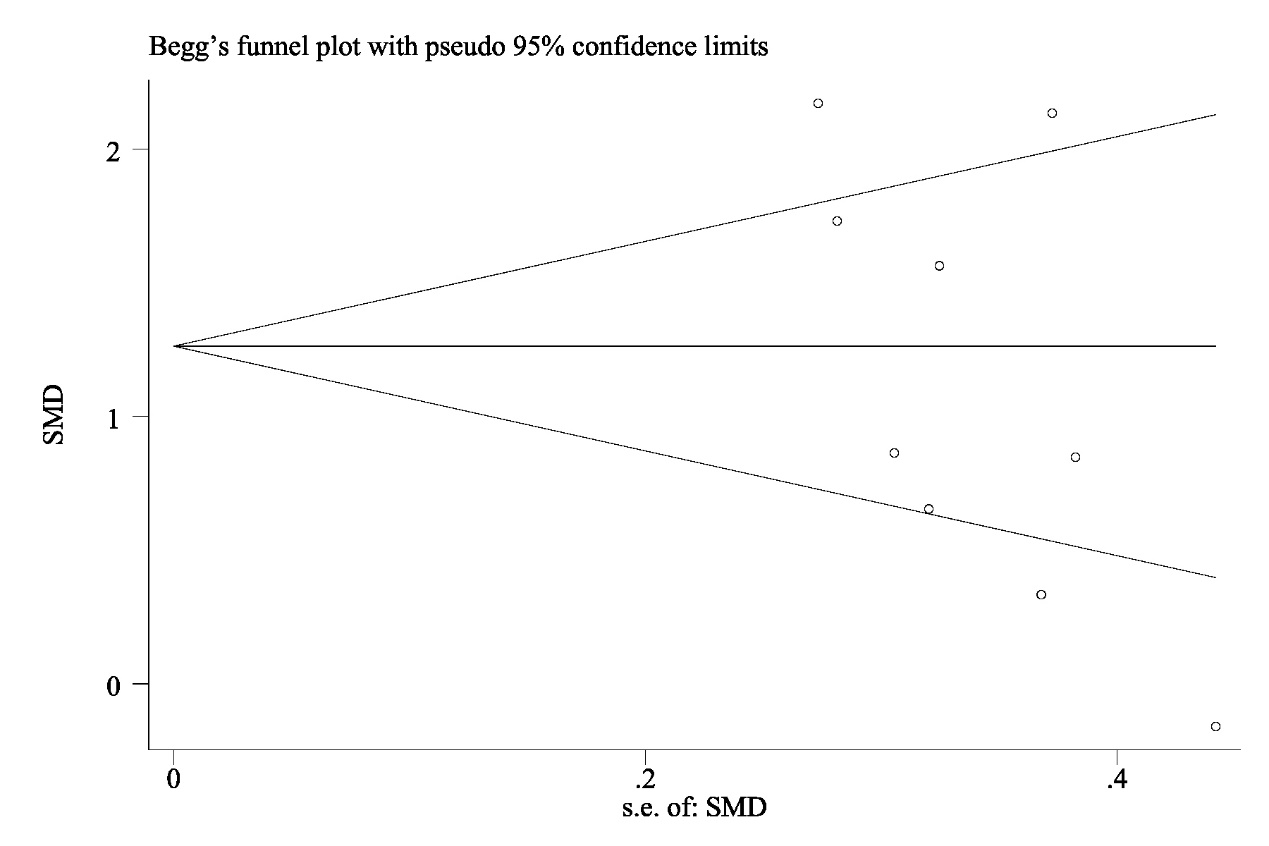


Fig S6 Step speed publication bias figure of included literature


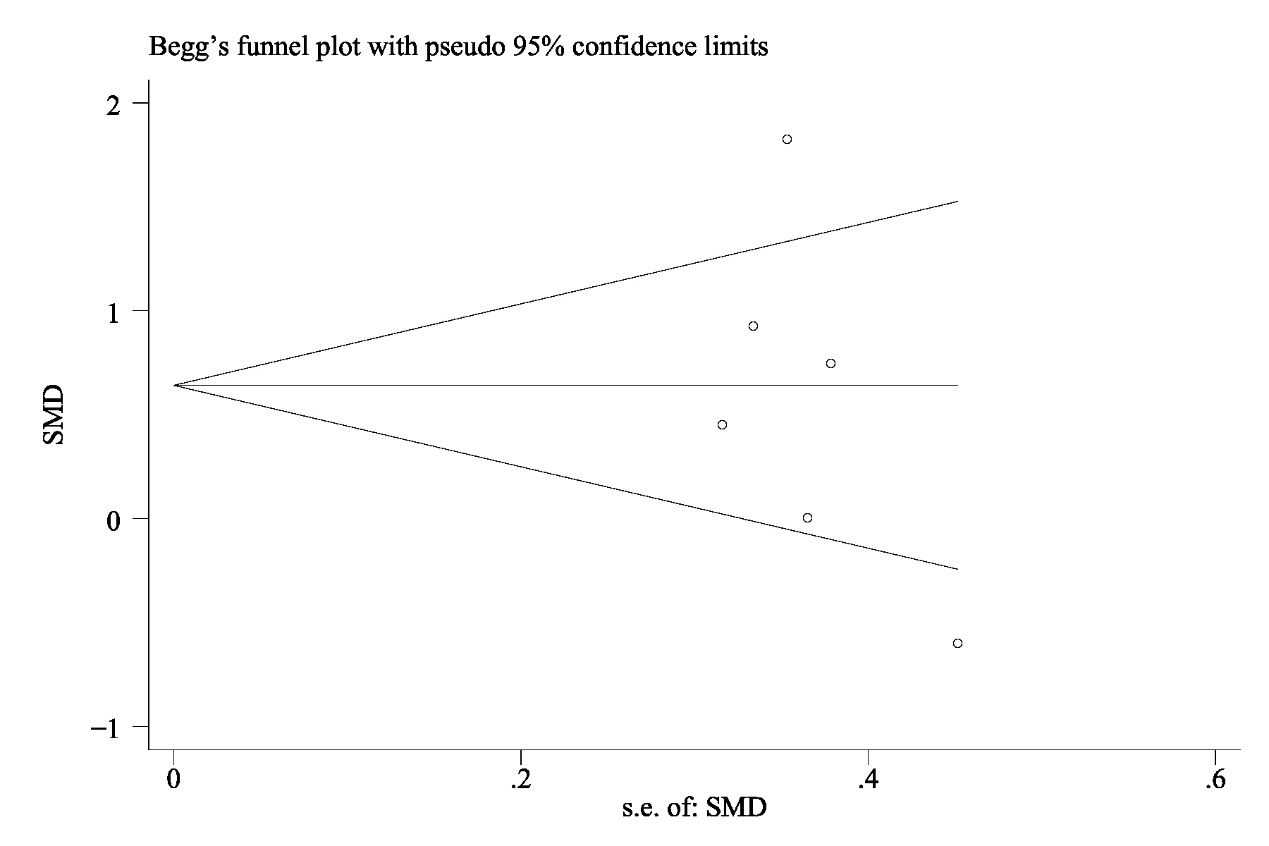


Fig S7 Cadence publication bias figure of included literature


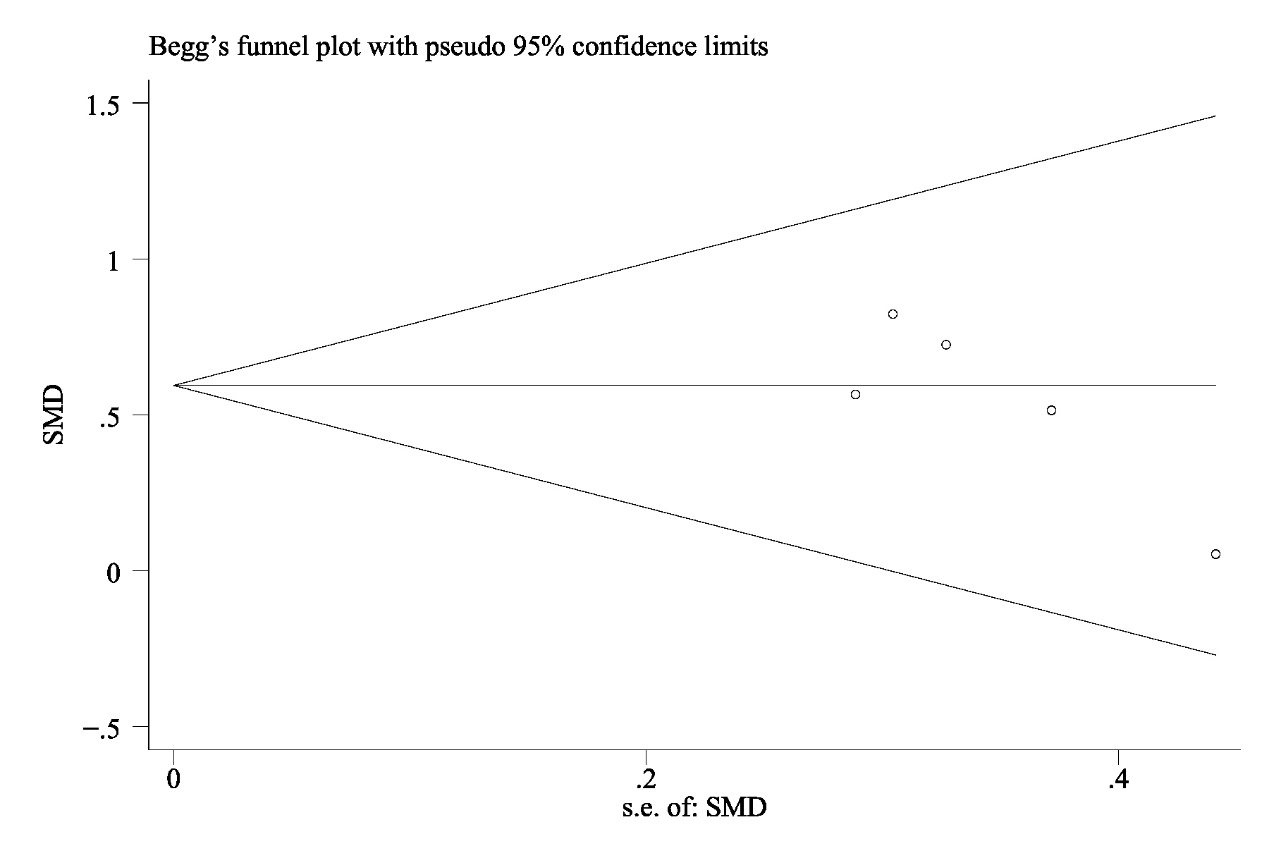


Fig S8 Stride length publication bias figure of included literature


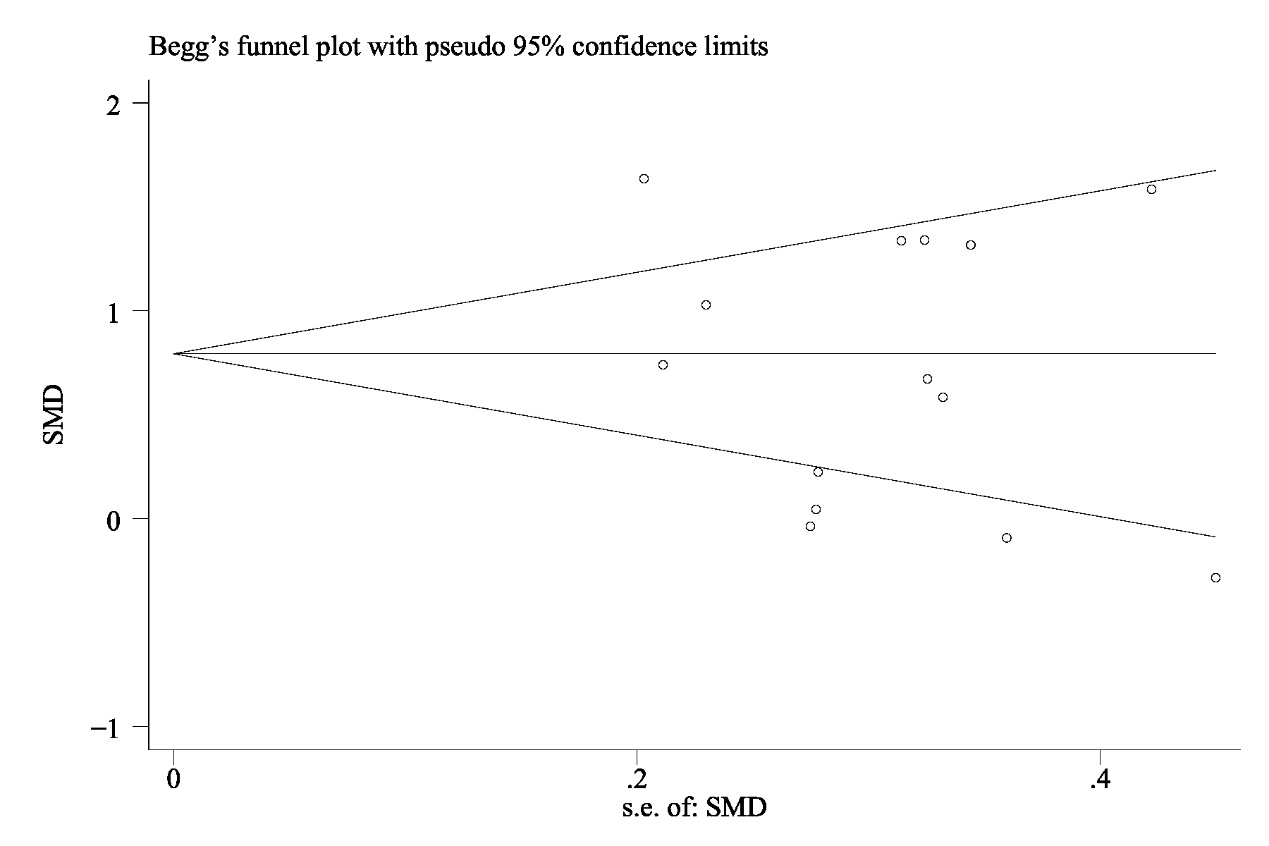


Fig S9 BBS publication bias figure of included literature


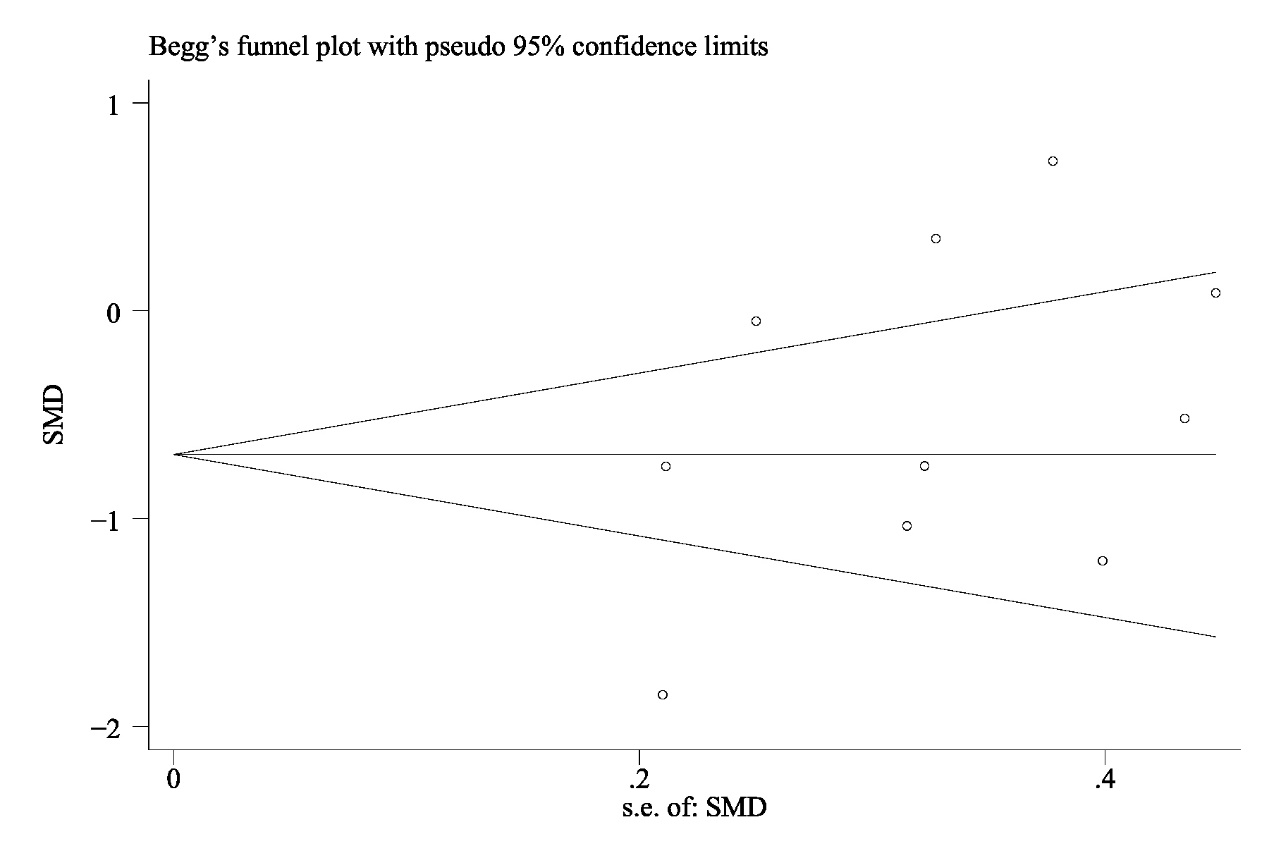


Fig S10 TUGT publication bias figure of included literature


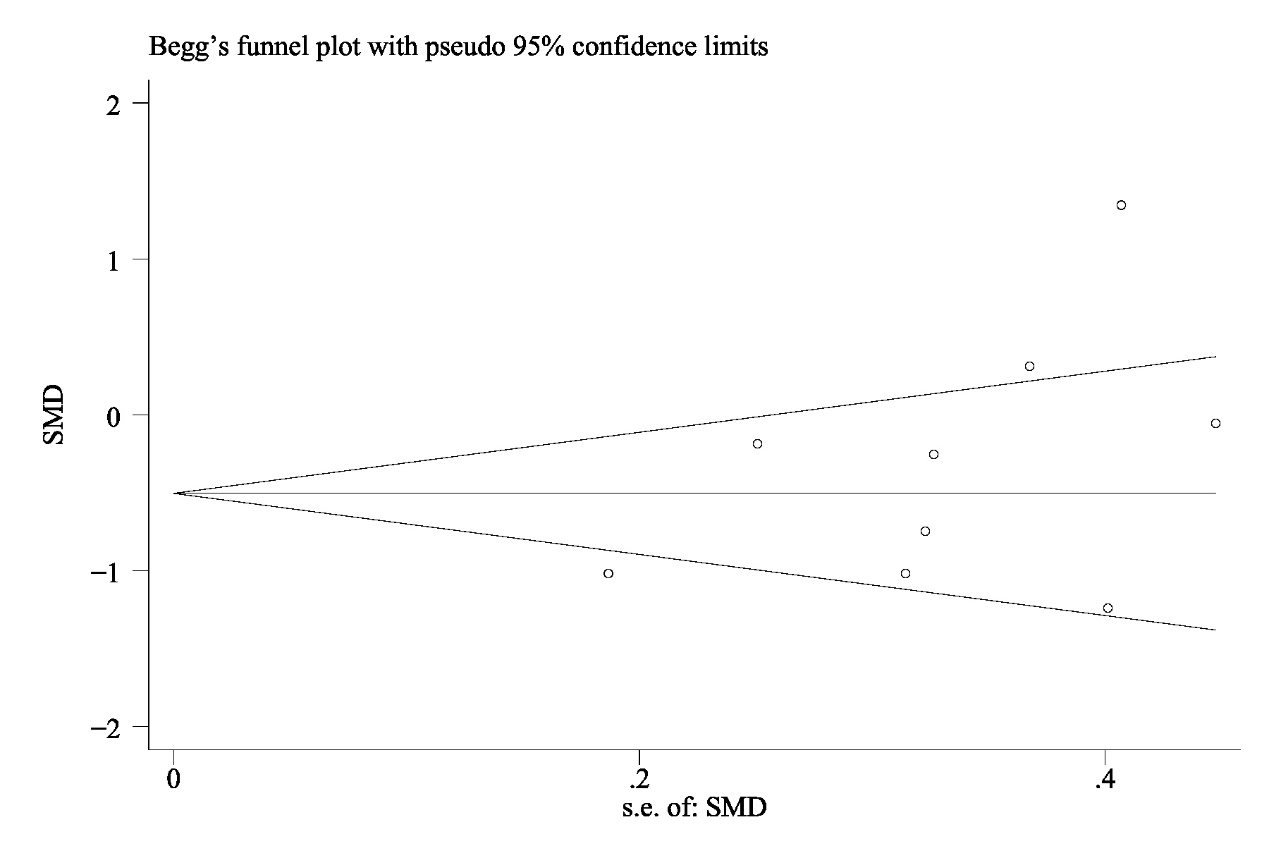


Fig S11 10MWT publication bias figure of included literature

## Supplementary Table

Supplementary Table 1.Search strategy for each database

| Search engine | Search query | Date of search |
| --- | --- | --- |
| CNKI/VIP/ CBM | #1 主题 (Topic) = 脑卒中 (stroke, cerebral apoplexy) OR 脑出血(encephalorrhagia) OR脑梗死(cerebral infarction)  #2 主题 (Topic) = 步行(walk) OR 步态(gait)  #3 主题 (Topic)= 全身振动训练(whole-body vibration training, WBVT) OR振动训练(vibration training,VT)  #4 #1 AND #2 AND #3 | 30-September-22 |
| Pubmed | #1 strokes[Title/Abstract]  #2 stroke[Title/Abstract]  #3 encephalorrhagia [Title/Abstract]  #4 cerebral apoplexy [Title/Abstract]  #5 CVA[Title/Abstract]  #6 cerebral hemorrhage[Title/Abstract]  #7 encephalorrhagia [Title/Abstract]  #8 #1 OR #2 OR #3 OR #4 OR #5 OR #6 OR #7  #9 walk [Title/Abstract]  #10 gait [Title/Abstract]  #11progression [Title/Abstract]  #12 #9 OR #10 OR #11  #13 whole-body vibration training[Title/Abstract]  #14 vibration training [Title/Abstract]  #15 vibration [Title/Abstract]  #16 VT [Title/Abstract]  #17 WBVT [Title/Abstract]  #18 #13 OR #14 OR #15 OR #16 OR #17  #19 #8 AND #12 AND #18 | 30-September-22 |
| Web of science | #1 TS = (‘strokes’ OR ‘stroke’ OR ‘encephalorrhagia’ OR ‘cerebral apoplexy’ OR ‘CVA’ OR ‘cerebral hemorrhage’ OR ‘encephalorrhagia’)  #2 TS = (‘walk’ OR ‘gait’ OR ‘progression’)  #3 TS = (‘whole-body vibration training’ OR ‘vibration training’ OR ‘vibration’ OR ‘VT’ OR ‘WBVT’)  #4 #1 AND #2 AND #3  Databases = SCI-EXPANDED, SSCI, A&HCI, CPCI-S, CPCI-SSH, ESCI | 30-September-22 |
| Embase | #1 ‘strokes’ OR ‘stroke’ OR ‘encephalorrhagia’ OR ‘cerebral apoplexy’ OR ‘CVA’ OR ‘cerebral hemorrhage’ OR ‘encephalorrhagia’  #2 ‘walk’ OR ‘gait’ OR ‘progression’  #3 ‘whole-body vibration training’ OR ‘vibration training’ OR ‘vibration’ OR ‘VT’ OR ‘WBVT’  #4 #1 AND #2 AND #3 | 30-September-22 |
| EBSCO | S1 strokes OR stroke OR encephalorrhagia OR cerebral apoplexy OR CVA OR cerebral hemorrhage OR encephalorrhagia  S2 walk OR gait OR progression  S3 whole-body vibration training OR vibration training OR vibration OR VT OR WBVT  S4 S1 AND S2 AND S3 | 30-September-22 |

Table note:CNKI:China National Knowledge Infrastructure; VIP:VIP database; CBM: China Biology Medicine disc;

| Reference | Generation of random sequences | randomization concealment | blind method | Withdrawal and withdrawal | score | level |
| --- | --- | --- | --- | --- | --- | --- |
| Qianhao et al., 2018 | 2 | 1 | 2 | 0 | 5 | H |
| Chuan et al., 2022 | 2 | 1 | 2 | 0 | 5 | H |
| Zhen-hua et al., 2019 | 2 | 2 | 2 | 0 | 6 | H |
| Xin-xin et al., 2020 | 2 | 1 | 2 | 0 | 5 | H |
| Yan et al., 2021 | 2 | 1 | 2 | 1 | 6 | H |
| Zhanyu et al., 2019 | 2 | 1 | 2 | 0 | 5 | H |
| Le et al., 2022 | 2 | 2 | 2 | 0 | 6 | H |
| Jin-Ming et al., 2021 | 2 | 1 | 2 | 0 | 5 | H |
| Zhenying et al., 2022 | 0 | 0 | 2 | 0 | 2 | L |
| van Nes et al., 2006 | 2 | 2 | 2 | 1 | 7 | H |
| Gu and Hwangbo, 2016 | 1 | 1 | 2 | 0 | 4 | H |
| Brogårdh et al., 2012 | 2 | 2 | 2 | 1 | 7 | H |
| Wei and Cai, 2022 | 2 | 2 | 2 | 1 | 7 | H |
| Ijaz Ahmed Burq et al., 2021 | 2 | 2 | 2 | 1 | 7 | H |
| Chan et al., 2012 | 2 | 2 | 2 | 1 | 7 | H |
| Sade et al., 2020 | 2 | 1 | 2 | 1 | 6 | H |
| Choi et al., 2016 | 2 | 0 | 2 | 0 | 4 | H |
| Kim and Lee, 2021 | 2 | 0 | 2 | 1 | 5 | H |
| Choi et al., 2017 | 2 | 2 | 2 | 1 | 7 | H |
| Lee, 2019 | 2 | 2 | 2 | 0 | 6 | H |
| Guo et al., 2015 | 2 | 1 | 2 | 0 | 5 | H |
| Xie et al., 2021 | 0 | 0 | 2 | 0 | 2 | L |

Supplementary Table 2. Jadad scale score of included literature

Table note:H: High quality;L:Low quality.
